# Nitrate of Silver as an Application to the Teeth

**Published:** 1854-04

**Authors:** B. T. Whitney

**Affiliations:** Buffalo, N. Y.


### *Nitrate of Silver as an application to the Teeth. By* Dr. B. T. WHITNEY, BUFFALO, N. Y.

There has long been, and is still, to a very great extent, a popular prejudice in the profession against the use of the nitrate of silver as a topical application to the teeth or mouth.

This arises, in part, from the known proportion of nitric acid used in its composition, or rather in its preparation, and the effect of acid upon the lime of the teeth, and, in part, from the coloring matter, supposing that disorganization of the tooth body must necessarily follow.

By many experiments, I perceive no chemical effect upon the body of the tooth, when immersed even in a saturated solution of nitrate of silver. This was anunexpected result; but when we look to the mode of the manufacture of the article, this result is clearly explained.

In the manufacture of the nitrate of silver, nitric acid and pure silver are alone employed, the acid being saturated by the silver. The liquid is then evaporated, and the nitrate remains in chrystals, which is heated to fusion in platina cups, and cast in silver molds.

Nitric acid is made by distilling a solution of nitrate of potash, saltpeter, and strong sulphuric acid, in the proportion of three parts of nitre to two of acid.

A simple statement of the bases and *modus operandi* of the manufacture of these articles, I conceive only to be necessary to enable any one to understand the point I intend to illustrate.

Nitric acid is partly decomposed by combination with any metalic oxide, and wholly by the application of heat, when the oxygen and nitrogen of which it is composed assume their gaseous state.

Now, in making nitrate of silver, both these means are employed—the metallic oxide and heat.

In fusing the chrystals, after the evaporation of the liquid, the acid is nearly decomposed, leaving the salts, holding the silver in chrystalization in the proportion of about 68 parts of the latter to 32 of the former.

The destructive effects of pure nitrate of silver upon animal tissue has brought it into use with the surgeon; but still, in a very weak solution, it preserves animal matter, and even prevents polished steel from tarnishing.

As an application to decayed or denuded teeth, that have become sensitive, I hold it in high estimation. It acts decidedly, and in a two-fold way—in destroying the animal fibers that, in their ramification through the body of the tooth, become exposed and inflamed—and then, by closing the mouth of the cells with the silver, which, in parting with its corrosive power, unites with oxygen, and forms an inert metallic oxyde. This gives a coating of insoluble metallic body over the denuded part of the tooth; though exceedingly thin, yet sufficient to protect the nervous filament and dentine from irritation and contact with the "outer world." The tooth body being porous, absorbs more or less of the nitrate, which soon oxydizes, and gives the tooth a blackened appearance. These canals, though sufficient to transmit nutriment from the nerve pulp, through the dentine, are too minute to allow the introduction of the particles of nitrate of silver to a very great depth, so that the discoloration is superficial.

That the oxyde of silver closes the cells, and forms a metallic surface, is perfectly demonstrable, by immersing a tooth, with the dentine exposed, in a solution of the nitrate, and then place it under the blow-pipe, with a heat sufficient to fuse the silver, when a bright silver surface will appear to the naked eye, susceptible of bearing a polish with a burnisher, almost equal to that deposited by the electro-galvanic battery upon a metal surface.

Teeth to which artificial substitutes are attached by clasps, usually suffer, either from decay or wearing away, and, sooner or later, become very tender to the the touch, or sensitive to changes of temperature. Nitrate of silver acts like a charm in allaying the tenderness, though it gives a black coating to the surface.

When used on these teeth, the plate should be left out for a few minutes, until the silver is oxydized, otherwise the clasp will also become discolored.

In denudation of the teeth—that is often very troublesome or in ordinary decay, it may be used freely without fear of injury.

The pure chrystals are much better than the common sticks. In the softening of a tooth under a clasp, I have obtained decided benefit from its free use, in preventing the destruction of the lime, and forming over the surface a hard and impervious coating, the semi-disorganized portion of the tooth absorbing a greater quantity of the silver, which, in oxydizing, becomes very hard.

Its effects, in most cases, in allaying tenderness of the dentine, is, of course, but temporary, as the coating of the oxyde is very light, and soon wears off, exposing the more highly organized part of the tooth to irritation.

Oft-repeated applications will usually prevent pain, and, in most cases, if not arrest, at least, greatly retard the injury to teeth from clasps or denudation.

*Buffalo, N. Y., Feb.*, 1854.

